# Identification of early diagnostic antigens from major excretory-secretory proteins of *Trichinella spiralis* muscle larvae using immunoproteomics

**DOI:** 10.1186/1756-3305-7-40

**Published:** 2014-01-22

**Authors:** Li Wang, Jing Cui, Dan Dan Hu, Ruo Dan Liu, Zhong Quan Wang

**Affiliations:** 1 Department of Parasitology, Medical College, Zhengzhou University, 40 Daxue Road, Zhengzhou 450052, P. R. China

**Keywords:** *Trichinella spiralis*, Muscle larvae, Excretory-secretory proteins, Early diagnostic antigens, Mass spectrometry

## Abstract

**Background:**

The excretory-secretory (ES) proteins of *Trichinella spiralis* muscle larvae (ML) come mainly from the excretory granules of the stichosome and the cuticles (membrane proteins), are directly exposed to the host’s immune system, and are the main target antigens, which induce the immune responses. Although the ES proteins are the most commonly used diagnostic antigens for trichinellosis, their main disadvantage are the false negative results during the early stage of infection. The aim of this study was to identify early specific diagnostic antigens from the main components of *T. spiralis* muscle larval ES proteins*.*

**Methods:**

Two-dimensional electrophoresis (2-DE) combined with Western blot were used to screen the early diagnostic antigens from the main components of *T. spiralis* muscle larval ES proteins. The protein spots recognized by the sera from BALB/c mice infected with *T. spiralis* at 18 days post-infection (dpi) were identified by MALDI-TOF/TOF-MS and putatively annotated using GO terms obtained from the InterPro databases.

**Results:**

The ES proteins were analyzed by 2-DE, and more than 33 protein spots were detected with molecular weight varying from 40 to 60 kDa and isoelectric point (pI) from 4 to 7. When probed with the sera from infected mice at 18 dpi, 21 protein spots were recognized and then identified, and they were characterized to correlate with five different proteins of *T. spiralis*, including two serine proteases, one deoxyribonuclease (DNase) II, and two kinds of trypsin. The five proteins were functionally categorized into molecular function and biological process according to GO hierarchy.

**Conclusions:**

2-DE and Western blot combined with MALDI-TOF/TOF-MS were used to screen the diagnostic antigens from the main components of *T. spiralis* muscle larval ES proteins. The five proteins of *T. spiralis* identified (two serine proteases, DNase II and two kinds of trypsin) might be the early specific diagnostic antigens of trichinellosis.

## Background

Trichinellosis is a widespread parasitic zoonosis caused by the ingestion of raw or inadequately cooked meat containing the infective larvae of the nematode genus *Trichinella*[1]. In the past few decades, many outbreaks of human trichinellosis have been reported in different areas of the world [2-4]. From 2004 to 2009, 15 outbreaks of human trichinellosis, consisting of 1 387 cases and 4 deaths, were reported in China [5]. So, trichinellosis is not only a public health hazard that affects patients but also represents an economic problem in porcine animal production and food safety [6].

The clinical diagnosis of trichinellosis is rather difficult because its clinical manifestations are nonspecific [7,8]. At present, muscle biopsy and serologic tests are used for diagnosing human trichinellosis [9,10]. The sensitivity of muscle biopsy depends on the amount of muscle sample tested and the degree of infection. Muscle biopsy is not sensitive to the light infections and the early stage of infection. Serologic tests [e.g., enzyme-linked immunosorbent assay (ELISA) using muscle larval excretory-secretory (ES) antigen or the synthetic antigen 3,6-dideoxy-D-arabinohexose (tyvelose)] for detecting the specific anti-*Trichinella* antibody IgG are not positive in pigs and mice infected experimentally until 3–4 weeks after infection [11-13]. The detection of IgG antibodies to *Trichinella* by ELISA using ES antigens of *T. spiralis* muscle larvae (ML) is commonly used for diagnosis of trichinellosis [9]. However, the main disadvantage of detection of IgG antibodies is the occurrence of a high rate of false negative results during the early stage of infection. Several studies have shown that the maximum detection rate of 100% of IgG antibodies was not reached until at least 1–3 months after human infection with the parasite [11,14]. The *T. spiralis* ML ES proteins were analyzed by SDS-PAGE or 2-DE gel analysis [15,16]. In the previous study, we attempted to identify early diagnostic antigens in *T. spiralis* ES proteins with molecular weights of 30–40 kDa [17]. However, The 40–60 kDa ES proteins of *T. spiralis*, which are the major antigenic proteins, have received more attention than other ES proteins because these proteins are candidates of immunodiagnostic antigens for trichinellosis and are present in much greater amounts in the ES products [18]. In this study, an attempt is made to screen early specific antigens from the most abundant proteins of *T. spiralis* ML ES proteins, which might be valuable for the early diagnosis of trichinellosis.

Immunoproteomics could be defined as the combination of any proteomic technology with an immunological data presentation [19]. Its development is vital in an age where it is increasingly becoming urgent to identify disease biomarkers and pathogenic target antigens for diagnosis and the development of new drugs and vaccines. Among the current proteomic techniques available [20], 2-DE has often been chosen as the research tool in immunoproteomic applications in combination with Western-blot [19]. In the present study, we identified the early specific diagnostic antigens from the main components of *T. spiralis* ML ES proteins using immunoproteomic methods.

## Methods

### Parasite and experimental animals

*T. spiralis* isolate (ISS534) used in this study was obtained from a domestic pig in Nanyang city of Henan Province, China. The isolate was maintained by serial passages in Kunming mice in our laboratory. Muscle larvae were recovered from the mice infected with *T. spiralis* at 42 dpi by artificial digestion as described previously [21,22]. Specific pathogen free (SPF) female BALB/c mice aged 6 weeks were purchased from the Experimental Animal Center of Henan province (Zhengzhou, China). The permission (No. SCXK 2010–0002) was given by the Science and Technology Department of Henan Province.

### Collection of infection sera and determination of anti-*Trichinella* antibodies

BALB/c mice were orally infected with 300 larvae/mouse and the serum samples from the infected mice were collected as described previously [23]. About 100 μl of tail vein blood was daily collected from each mouse before infection and during 14–42 dpi, respectively. The anti-*Trichinella* IgG antibodies of infected mice during 14–42 dpi were determined by the ELISA and Western blot methods using *T. spiralis* ML ES proteins as antigens. The specific antibodies were firstly detected at 18 dpi by the above-mentioned two methods and persisted to 42 dpi (data not shown). The early (18 dpi) and late (42 dpi) infection sera were used to detect the fractions of ES proteins in the following Western blot analysis.

### Preparation of ES proteins

Preparation of ES proteins was performed as previously described [24,25]. Briefly, after being washed thoroughly in sterile saline and serum-free RPMI-1640 medium supplemented with 100 U penicillin/ml and 100 μg streptomycin/ml, the larvae were incubated in the same medium at 5 000 worms/ml for 18 h at 37°C in 5% CO_2_. After incubation, the media containing the ES proteins were poured into 50-ml conical tubes and the larvae were allowed to settle for 20 min. The supernatant containing the ES products was filtered through a 0.2 μm membrane. The ES products were dialyzed and then lyophilized by a vacuum concentration and freeze-drying (Heto Mxi-Dry-Lyo, Denmark). The protein concentration was determined by the Bradford assay [26].

### 2-DE and Western blot

The ES proteins were precipitated using trichloroacetic acid (TCA) and acetone using a previously described method with some modifications [27]. The electrophoresis was performed as described previously [17,28]. Briefly, 300 or 200 μg of ES proteins were loaded onto 11-cm pH 4–7 immobilized pH gradient (IPG) strips (Bio-Rad, USA) and separated by isoelectric focusing (IEF). IEF was performed using a Protean IEF Cell at 20°C as follows: S1: 50 V, 12 h; S2:250 V, 30 min; S3: 1 000 V, 30 min; S4: 8 000 V, 4 h; and S5: 8 000 V, 40 000 Vh (using a limit of 50 μA/strip). SDS-PAGE was performed with 10% gels using a Mini Protean cell (Bio-Rad, USA). Three replicates were run for the sample. After 2D gel electrophoresis, proteins were either stained with Coomassie blue R-250 for proteomic analysis or used for immunoblotting as previously described [29]. Both the 2-DE and immunoblotting tests were repeated three times, with no variation in results observed. Images of immunoblots were captured using ImageScanner (GE healthcare, USA) and aligned with equivalent protein stained 2-DE gels using Image Master 2D Platinum 6.0 (GE healthcare, USA).

### Protein identification and database searches

2-DE gel excision and tryptic digestion of 2-DE gel electrophoresis protein spots recognized by early infection sera were prepared for MALDI-TOF/TOF-MS analysis according to standard protocols [30]. The resulting peptides were analyzed by MALDI-TOF/TOF-MS. The procedure was performed as described previously [31]. Combined peptide mass fingerprinting (PMF) and MS/MS queries were performed by using the MASCOT search engine 2.2 (Matrix Science, UK) and submitted to MASCOT Sequence Query server (http://www.matrixscience.com) for identification against nonredundant NCBI database (http://www.ncbi.nlm.nih.gov/BLAST). The criteria for successfully identified proteins were as follows: ion score confidence index (CI) for peptide mass fingerprint and MS/MS data was ≥95%.

### InterPro annotation and gene ontology (GO) categories

Functional characterization of *Trichinella* protein sequences was based on Gene Ontology (GO) Annotation. InterProscan software (http://www.ebi.ac.uk/Tools/pfa/iprscan/) was used to perform protein sequence searches against InterPro member databases to identify signatures [32]. The resultant proteins were functionally categorized using the Web Gene Ontology Annotation Plot [WEGO, (http://wego.genomics.org.cn/cgi-bin/wego/index.pl)] [33]. The groups of datasets were simultaneously subjected to online analysis that was convenient to compare them in one graph.

### Ethics statement

All animals were treated in strict accordance to the National Guidelines for Experimental Animal Welfare (MOST of People’s Republic of China, 2006). The protocols of the animal experiments reported herein were approved by The Life Science Ethics Committee of Zhengzhou University.

## Results

### 2-DE analysis of *T. spiralis* ES proteins

**Figure 1:**
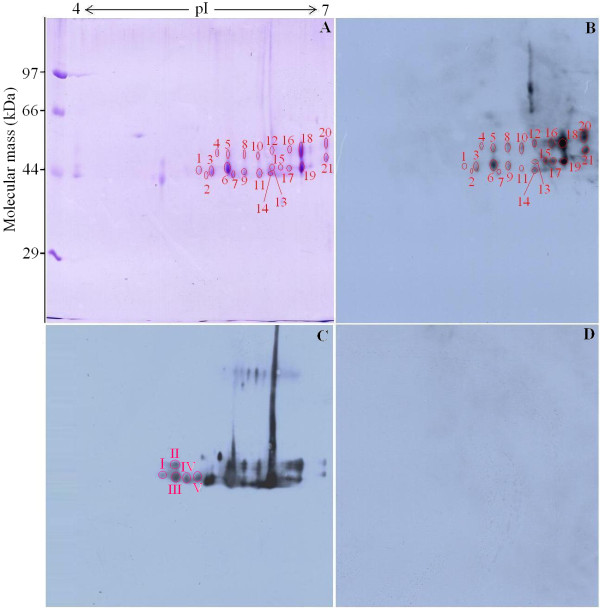
**2-DE and Western blot analysis of *Trichinella spiralis *muscle larval major excretory-secretory (ES) proteins. (A)** 2-DE gel of major ES proteins separated in the first dimension in the pH range 4–7 and then in the second dimension on a 10% polyacrylamide gel. The gel was stained with Coomassie blue R-250, molecular weight standard is on the left, and pI values are indicated. Protein spots selected for analysis are numbered. **(B)** 2-DE Western blot of major ES proteins probed by mouse infection sera at 18 days post infection (dpi), and the immunoreactive protein spots were detected by the enhanced chemiluminescent (ECL) kit. **(C)** 2-DE Western blot of major ES proteins probed by mouse infection sera at 42 dpi, I-V was the additional 5 positive spots recognized only by infection sera at 42 dpi. **D)** Western blot map probed by sera from normal mice before infection.

With the purpose of improving the resolution of the spots, IEF was performed in pH 4–7 narrow range strips in this study. Firstly, the second dimension (SDS-PAGE) was performed using 12% polyacrylamide gels. After separation, the 2-DE gel was stained with Coomasie blue R-250, and the main components (43, 45, and 53 kDa proteins) of ES proteins were aggregated, which are present in much greater amounts. For better separation of the most abundant proteins, the load amount of ES protein was reduced from 300 to 200 μg and SDS-PAGE was performed using 10% polyacrylamide gels, where the majority of the main components of ES proteins are present but some proteins with low molecular weight disappeared. After IEF in pH 4–7 linear IPG strips and separation by 10% SDS-PAGE with 200 μg of ES proteins, more than 33 spots were detected, with molecular weight from 40 to 60 kDa (Figure 1A). The 2-DE was repeated three times, and the patterns were highly reproducible.

### Western blot analysis of *T. spiralis* ES proteins following 2-DE

As shown in Figure 1B, there were more than 25 spots displaying reactivity with the infection sera at 18 dpi. Once photographed, the immunoblot and their homologous Coomassie blue-stained gel were aligned and then matched by Image Master 2D Platinum 6.0 software and artificial recognition. A total of 21 immunoreactive protein spots could be confidently matched to the corresponding protein spot in Coomassie blue-stained gels. These matched spots named spot 1 to 21 were selected to be further analyzed by MS. In comparison, when the immunoblot was performed with sera at 42 dpi, there were the additional 5 positive spots (Figure 1C) more than the above-mentioned 25 spots, indicating that the 25 positive spots were recognized by both infection sera at 18 dpi and 42 dpi, but the stronger reactions were observed with sera at 42 dpi, and the additional 5 positive spots were recognized only by infection sera at 42 dpi. But, the sera collected from normal mice before infection did not show detectable immunoreactivity with any of the protein spots (Figure 1D).

### Identification of immunoreactive proteins by MALDI-TOF/TOF-MS

Out of all 21 matched protein spots recognized by infection sera at 18 dpi, 17 spots were successfully identified. Of these, 16 spots were characterized to correlate with five different proteins of *Trichinella*, including two serine proteases with different pI and molecular weight values, one deoxyribonuclease (DNase) II, and two kinds of trypsin, while the other spot was identified as alpha S1 casein of *Bos Taurus.* The proteins of other 3 spots were identified as protein of other organism species and not matched with that of *Trichinella* in the NCBI database. Although another protein spot was matched with *Trichinella* protein, the MOWSE score was only 54, which was unsuccessfully identified. The results of protein identification are shown in Table 1.

**Table 1:** **Identification of *Trichinella spiralis *** **muscle larval major ES proteins recognized by mouse infection sera at 18 dpi using MALDI-TOF/TOF-MS**

**Spot no.**	**Protein name**	**Species**	**Accession no.**	**Theoretical Mr/pI**^**a**^	**MOWSE score**^**b**^	**Coverage (%)**	**No. matched peptides**	***E*-value**
1	Serine protease	*T. spiralis*	gi|168805931	35.7/5.97	335	22	8	7e-027
2	Alpha S1 casein	*Bos taurus*	gi|159793193	18.7/5.23	208	53	8	3.5e-014
3	Putative trypsin	*T. spiralis*	gi|339241891	31.3/5.25	132	21	3	1.4e-006
4	Unknown	*Legionella rubrilucens*	gi|61814442	12.0/6.84	43	61	5	1e + 003
5	Putative trypsin	*T. spiralis*	gi|339241897	53.9/5.97	308	17	8	3.5e-024
6	Serine protease	*T. spiralis*	gi|168805931	35.7/5.97	608	28	6	3.5e-054
7	Deoxyribonuclease II family protein	*T. spiralis*	gi|339241449	38.1/5.97/	440	30	8	2.2e-037
8	Serine protease	*T. spiralis*	gi|13641204	48.7/6.33	337	18	8	4.4e-027
9	Serine protease	*T. spiralis*	gi|13641204	48.7/6.33	200	11	3	2.2e-013
10	Serine protease	*T. spiralis*	gi|13641204	48.7/6.33	405	17	7	7e-034
11	Serine protease	*T. spiralis*	gi|13641204	48.7/6.33	347	15	6	4.4e-028
12	Deoxyribonuclease II family protein	*T. spiralis*	gi|339241449	38.1/5.95	195	28	7	7e-013
13	Deoxyribonuclease II family protein	*T. spiralis*	gi|339241449	38.1/5.95	336	34	10	5.5e-027
14	Hypothetical protein	*Afipia felis*	gi|414163749	35.7/10.2	56	36	9	54
15	Hypothetical protein	*Pantoea ananatis*	gi|378767336	35.1/10.9	49	100	4	2.7e + 002
16	Serine protease	*T. spiralis*	gi|13641204	48.7/6.33	336	17	8	5.5e-027
17	Serine protease	*T. spiralis*	gi|13641204	48.7/6.33	54	14	5	94
18	Serine protease	*T. spiralis*	gi|13641204	48.7/6.33	304	11	4	8.8e-024
19	Serine protease	*T. spiralis*	gi|13641204	48.7/6.33	443	22	6	1.1e-037
20	Serine protease	*T. spiralis*	gi|168805933	48.7/6.33	353	11	9	1.1e-028
21	Serine protease	*T. spiralis*	gi|168805933	48.7/6.33	471	20	9	1.8e-040

^a^Theoretical molecular mass (kDa) and isoelectric point (pI).

^b^A MOWSE score of >70 was used to assign identity to a protein.

### Functional categorization of immunoreactive proteins by gene ontology

**Figure 2:**
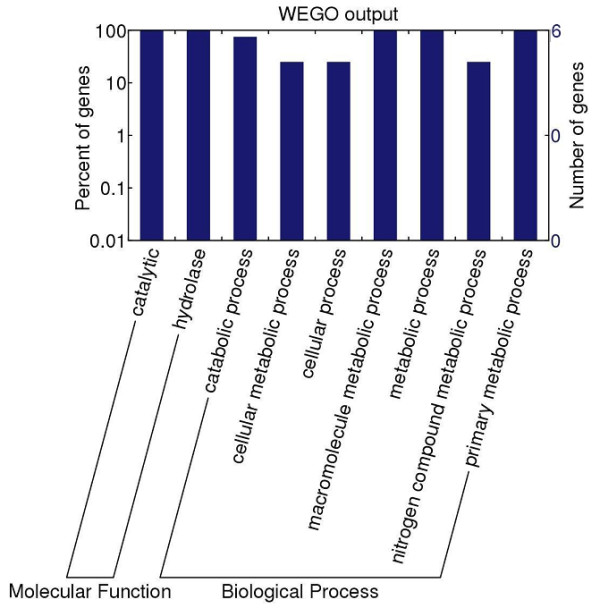
**Gene ontology (GO) categories of *Trichinella spiralis *muscle larval major excretory-secretory (ES) proteins recognized by mouse infection sera at 18 days post infection (dpi).** The identified proteins were classified into molecular function and biological process by WEGO according to their GO signatures. The number of genes denotes that of proteins with GO annotations.

To further understand the functions of the proteins identified in this study, the identified 5 protein sequences were putatively annotated using GO terms obtained from the InterPro databases. In addition, the 5 proteins were functionally categorized into molecular function and biological process according to GO hierarchy using WEGO. The classification results of the 5 proteins identified in this study were shown in Figure 2. For the molecular function category, five proteins had catalytic and hydrolase activity. In the biological process category, all of the 5 proteins were also associated with metabolic process.

## Discussion

Parasites are designed by evolution to invade and survive in hosts; they release a variety of molecules that help them to penetrate the defensive barriers and avoid the immune attack of the host [34]. Modulation of the immune response by helminthes involves the ES proteins released by these parasites, including proteases, protease inhibitors, allergen homologues, glycolytic enzymes, lipids and glycans [35,36]. The ES antigens of *Trichinella* spp. can induce a strong immune response involving the generation of specific antibodies and may be very important for serodiagnosis as they are easily targeted by the host’s immune system [37].

In our previous study, after IEF in pH 3–10 nonlinear IPG strips and separation by 12% SDS-PAGE, the 2-DE gel was stained with Coomasie blue R-250, and most of the protein spots were located between pH 4 and 7 [17]. With the purpose of improving the resolution of the spots, IEF was performed in pH 4–7 narrow range strips in this study. Previous studies demonstrated that all true positive sera (i.e., sera from persons with confirmed trichinellosis as well as sera from naturally and experimentally infected pigs), reacted with a three-band pattern ranging in size from 48–72 kDa using ES antigens of *T. spiralis* ML [38]. The 40–60 kDa ES proteins of *T. spiralis*, which are the major antigenic proteins, have received more attention than other ES proteins because these proteins are candidates of immunodiagnostic antigens for trichinellosis and are present in much greater amounts in the ES products [18]. Hence, in this study, we screened the early specific antigens from the main components of ES proteins with 40–60 kDa, which might be valuable for the early diagnosis of trichinellosis. Our results showed that the 21 protein spots were recognized by mouse infection sera at 18 dpi and analyzed by MALDI-TOF/TOF-MS. Out of the 21 protein spots, 16 spots were successfully identified and represented only five different proteins of *Trichinella*, and all of them were identified to protease: two kinds of serine protease [SP-1.2(gi|168805931), SP-1.3(gi|13641204)] and two kinds of trypsin (gi|339241891 and gi|339241897) belong to the trypsin-like serine protease (Tryp_SPc) superfamily, while the DNase II (gi|339241449) belongs to phospholipase D (PLD_SF) superfamily. Indeed, by comparing our data with the previously published data of the ES proteins with 30–40 kDa recognized by early infection sera using the same approach [17], we found that three kinds of proteins (SP-1.2, SP-1.3, and DNase II) identified in both studies were the same proteins. However, the two studies are complementary inasmuch as two kinds of proteins identified in this study were not identified in our previous study. Combined, both studies have identified seven kinds of proteins from the ES products recognized by early infection sera.

Several experiments have shown that proteolytic enzymes are present in the ES products of *T. spiralis* ML [39,40]. Several proteases (such as serine and cysteine) have been identified by substrate gel electrophoresis and characterized according to their pH optima, substrate specificities and inhibitor sensitivities [31,41]. Moreover, protease can serve as an immunodominant antigen, stimulating a protective immune response [42,43]. In this study, all the five different proteins are identified as protease, which may be synthesized as inactive precursor zymogens that are cleaved during limited proteolysis to generate their active forms.

A previous study has also demonstrated that *T. spiralis* may express more than one isoform of the protein and that a common precursor protein could undergo variable post-translational processing [41]. In this study, three different proteins (SP-1.2, SP-1.3, and DNase II) are identified in multiple protein spots. The results suggested that these proteins might have different isoforms of the same protein, or that some of these proteins might be processed by alternative splicing, post-translational modifications and protein processing [31,44]. These modifications could be related to phosphorylation or acetylation of the proteins after translation, and they could be vital for the protein’s biological functions, such as parasite survival, immune escape and immunopathogenesis. Three spots (7, 12, and 13) were identified as DNase II, which may have DNase IIα activity. DNase IIα is an acidic endonuclease and was found in lysosomes and nuclei [45]. The secretion of DNase II by *T. spiralis* may enhance the degradation of released DNA that had not yet been cleared by phagocytes, which may have evolved to affect the availability and down-regulation of host inflammatory processes [46]. Furthermore, this prediction is supported by the evidence that there are a variety of other nucleotide-metabolizing enzymes in *T. spiralis* that were postulated to suppress the proinflammatory effects of these nucleotides [47]. Moreover, 4 protein spots were identified as protein of other organism species and not matched with that of *Trichinella* in the NCBI database. Spot 2 was identified as alpha S1 casein of *Bos taurus*, which belongs to the alpha-casein family and plays an important role in the capacity of milk to transport calcium phosphate. Spot 14 was identified as hypothetical protein of *Afipia felis*, which contains one HTH lysR-type DNA-binding domain. Spot 4 was matched to an unknown protein of *Legionella rubrilucens* while spot 15 was identified as hypothetical protein of *Pantoea ananatis*. Besides, twelve spots (1, 6, 8, 9, 10, 11, 16, 17, 18, 19, 20 and 21) are matched to TspSP-1, but they have the different MW and pI. Multigene protease families have been found in numerous parasites such as *Fasciola hepatica*[48], *Haemonchus contortus*[49] and *Aspergillus flavus*[50]. Each member of the protease families may have a specific role dependent on particular regulation (constitutive or stage-specific), location (intestinal tract or excreted), the presence of a regulatory domain (in N or C terminal part) or variations in the amino acid sequence [51]. The above-mentioned five proteins from *T. spiralis* major ES proteins recognized by early infection sera might be the early specific diagnostic antigens for trichinellosis, which needs to be confirmed in further experiments.

## Conclusions

In this study, 2-DE and Western blot combined with MALDI-TOF/TOF-MS was used to screen the diagnostic antigens from the main components of *T. spiralis* muscle larval ES proteins. The main components of *T. spiralis* muscle larval ES proteins recognized by early infection sera were analyzed by MALDI-TOF/TOF-MS. The five *T. spiralis* proteins identified (two serine proteases, DNase II and two kinds of trypsin) might be the early specific diagnostic antigens of trichinellosis. The results suggest that immunoproteomics is a useful approach to identify the early diagnostic antigens.

## Competing interests

The authors declare that they have no competing interests.

## Authors’ contributions

ZQW and JC conceived and designed the experiments. LW, JC, DDH, RDL and ZQW performed the experiments. LW, JC and ZQW, analyzed the data and wrote the manuscript. All authors read and approved the final version of the manuscript.
